# Concomitant *Plasmodium falciparum* and intestinal helminth infections in a rural community of southern Côte d’lvoire

**DOI:** 10.1186/1475-2875-11-S1-P105

**Published:** 2012-10-15

**Authors:** Richard B Yapi, Eveline Hürlimann, Kigbafori D Silué, Clarisse A Houngbedji, Chammartin Frédérigue, Penelope Vounatsou, Jürg Utzinger, Eliezer K N’Goran, Giovanna Raso

**Affiliations:** 1 Departement Environnement et Santé, Centre Suisse de Recherches Scientifiques, Abidjan, Côte d’lvoire, 01 BP 1303 Abidjan 01; 2 Unité de Formation et de Recherches en Biosciences, Université de Cocody, Abidjan, Côte d’lvoire, 22 PB 582 Abidjan 22; 3 Department of Epidemiology and Public Health, SwissTropical and Public Health Institute, Basel, P.O 4002 Basel, Switzerland; 4 University of Basel, P.O 4003 Basel, Switzerland; 5 Unité de Formation et de Recherche en Biologie de la reproduction Animale, Université d’Abobo-Adjamé, Abidjan, Côte d’lvoire, 01 BP 801 Abidjan 01

## Background

Despite efforts to control the disease, malaria is still threatening the life of millions of people in sub-Saharan Africa [1]. In addition the distribution of malaria often overlaps in space with so called neglected tropical diseases (NTDs). People in endemic areas can therefore host more than one parasite species infection at the same time, hence making polyparasitism a common phenomenon [2-4]. The consequences of these diseases are manifold and can include impairment of cognitive development and anemia, school aged-children and pregnant women representing the most vulnerable groups with particular risk of morbidity [4-6]. In Côte d’lvoire, these diseases are widely prevalent but vary in their spatial distribution and present different patterns of associations. Risk factors such as distance to water bodies and socio-economic status have been identified among the underlying causes for this heterogeneity [7,8] As a result of this heterogenous occurrence of multi-parasite infections related morbidity and burden due to polyparasitism will vary as well between areas. For control activities decision making usually takes place at global and/or national level but for integrated, cost-effective and sustainable control efforts better understanding of co-infection dynamics at different spatial scales are urgently needed. The main goal of this study was therefore to describe the pattern of concomitant infections with *Plasmodium* and intestinal helminths in a rural setting in southern Côte d’lvoire.

## Method

A cross-sectional study was conducted in a hamlet of Azaguié, named Ancien Carrefour”, located 40 km from Abidjan, in September 2011. Blood and faecal specimens were collected to identify *Plasmodium spp*, *Schistosoma mansoni*, soil-transmitted helminths (hookworm, *Ascaris lumbricoides*, *Trichuris trichiura*), and intestinal protozoan infections by microscopy using standardized quality-controlled procedures. The study involved 413 persons from 85 households. Data analysis was done using logistic and multinomial regression models taking into account household effects.

## Results

*Plasmodium falciparum* overall prevalence was 60.53% (Table 1), which means a parasitemic index (PI) of 57.55% that characterises a hyper endemic malaria area. Predominant NTD parasites *were Schistosoma mansoni* (27.36%) and hookworm ((31.23%) (Table 1). These parasites overlapped with *P. falciparum*. The co-infection prevalences *of P. falciparum-S. mansoni* and *P. falciparum*-hookworm were 15.98% and 18.16%, respectively. Participants older than 5 years are at higher risk of co-infection compared to their younger. Multinomial analysis of co-infection of *P*. *falciparum-S. mansoni* (Table 2) reflected no significant association of age and sex to the co-infection risk. However, age was negatively related to the *P. falciparum* monoinfection risk, while female and age were negatively associated to the *S. mansoni* mono-infection risk. Multinomial analysis of co-infection of *P.falciparum*-hookworm (Table 3) showed that female were positively associated to the co-infection risk, while female and age presented a positive association to the hookworm mono-infection risk and age presented a negative association to *P. falciparum* mono-infection risk.

**Table 1:** Logistic regression for single parasite species infections regardless of any other parasite species infection, accounting for household effects

Parasite	People infected (%)	Independent variable	People infected (%)	OR (95%CI)^a^	P -value
*P. falciparum*	250 (60.53)	Age (years)			
		0-5	57 (68.67)	1	
		5-16	96 (82.05)	2.08 (1.06;4.08)	0.032
		>16	97 (45.54)	0.38(0.23;0.63)	<0.001
		Sex			
		Female	1 29 (60.28)	1	
		Male	121 (60.80)	1.03(0.68;1.58)	0.872
*S. mansoni*	113 (27.36)	Age (years)			
		0-5	1 (1.20)	1	
		5-16	28 (23.93)	25.80(3.08 ;173.73	0.001
		>16	84 (39.44)	53.81(7.21 ;401.50	<0.001
		Sex			
		Female	28 (22.43)	1	
		Male	65 (32.66)	1.69(1.10;2.60)	0.016
*Hookworm*	129 (31.23)	Age (years)			
		0-5	4 (4.82)	1	
		5-16	39 (33.33)	9.88(3.50;27.80)	<0.001
		>16	86 (40.38)	13.21(4.06;34.50)	<0.001
		Sex			
		Female	54 (25.23)	1	
		Male	75 (37.69)	1.77(1.23;2.54)	0.002

^a^Odds Ratio

**Table 2:** Multinomial logistic regression for *P. falciparum* and *S. mansoni* mono and co-infection with age and sex as independent variables, accounting for household effects

		Covariates			
	
		Sex		Age	
		
Infection	Positive for infection (%)	RRR(CI95% Cl)^b^	P-value	RRR(CI95% Cl)^b^	P-value
*P. f* mono-infection**	140 (33.90)	1.2(0.71 ;2.02)	0.495	0.96(0.95;0.98)	<0.001
*S.m* mono-infection**	20 (4.84)	2.31 (1.21;4.40)	0.011	1.01(1.00;1.03)	0.102
*P.f-S.m* co-infection	35 (8.47)	1.77 (0.91 ;3.42)	0.092	0.99(0.98;1.01)	0.421

^b^ Relative risk ratio

**People infected only with this parasite

**Table 3:** Multinomial logistic regression for *P. falciparum* and Hookworm mono and co-infection with age and sex as independent variables, accounting for household effects

		Covariates			
	
		Sex		Age	
		
Infection	Positive for infection (%)	RRR(CI95% CI)^c^	P-value	RRR(CI95% CI)^c^	P-value
*P. f* mono-infection**	140 (33.90)	1.16(0.68;1.98)	0.581	0.97(0.95;0.99)	<0.001
Hookworm mono-infection**	27 (6.54)	2.1(1.09;4.05)	0.026	1.02(1.01;1.04)	0.102
*P.f-Hookworm* co-infection	44 (10.65)	1.77(1.01 ;3.09)	0.045	0.99(0.98;1.01)	0.038

^c^ Relative risk ratio

**People infected only with this parasite

## Conclusion

This study confirms that polyparasitism is common in rural settings. However, implications of polyparasitism on morbidity and quality of life are not well understood. Further research should focus on understanding co-infection dynamics on the purpose of designing and implementing a sustainable intregreted control strategy.
